# Painful Affections

**Published:** 1873-04

**Authors:** 


### PAINFUL AFFECTIONS.

Persons sometimes suffer a variety of pains, more or les permanent, without being able to trace out the cause, the bodily functions seeming to be in healthy action, and yet medicine has no efficiency towards their removal. Sometimes these pains come on at regular intervals; at others they appear to depend on the state of the weather, or time, of day, or age of the moon, or season of the year. Grateful and permanent relief, more or less prompt, is sometimes experienced on discarding the use of some accustomed stimulant, in the form of tobacco, snuff, cigars, tea, coffee-bitters, or liquors; all these things stimulate the brain, and if their use is continued the constant stimulation ends in inflammation, communicating itself to the whole nervous system, causing a diseased condition of every fibre of the human body.

In many cases a single stimulation of the brain or nervous system is a medication of value and virtue, but habitual stimulation is always pernicious, because it occasions increased action, an action which is contrary to nature, which nature never intended, never made provision for, hence must be hurtful. When a man feels the need of a stimulant, it really means that the system wants rest, the mind repose, and the brain sleep. Rest and sleep are the only safe stimulants, because they bring back to the system its natural power of action. Sleep and rest are, to the body and brain, what letting down the water gate is to the old fashioned mill at night; the water accumulates by the morning to be used during the day; so the brain power and the nervous energy, by which we work and think, are recuperated, are accumulated during the night by sleep and rest. If the brain is never stimulated, is never recuperated, except by natural healthful sleep, as the result of temperate living and moderate industries, there is no reason in the world why the mind should not be as active and vigorous and efficient at fourscore as at forty. Therefore, if a person suffers from an obscure cause let him leave off any accustomed artificial stimulus, and in return or in place of it secure a larger supply of nature's stimulus, that of rest to the body, and sleep to the brain ; but rest to the body does not mean cessation from all exercise or work, for the best method in the world to enable persons to sleep, who are so unfortunate as to have to complain every morning of

POOR SLEEP, is enough bodily exercise in the open air every day, moderate exercise—slow rather than fast, long continued rather than short and violent ; such exercise as makes one a little tired, with an interested object in view, something desirable or important to be accomplished ; this is the only natural soporific. Now and then, for a night or two, it may be essential to cause sleep by the use of some form of medicine, but to use it for that purpose day after day, week after week, is perfectly ruinous to mind, body and disposition. A grand truth is involved in a single saying of the Scriptures, "The sleep of a laboring man is sweet," and he thrives upon it. That from opium and chloral and morphia is wormwood in the end.

